# Supplementary material for: Conformational variability of HIV-1 Env trimer and viral vulnerability
Source: eLife. 2026 Jun 26;15:RP110107. doi: 10.7554/eLife.110107 (PMC13309128; doi:10.7554/eLife.110107)
Supplement: Supplementary file 1. [file elife-110107-supp1.docx]

Supporting Information

**Conformational Variability of HIV-1 Env Trimer and Viral Vulnerability**

Yiwei Cao^1^ and Wonpil Im^1^*

^1^Department of Biological Sciences, Lehigh University, 111 Research Dr, Bethlehem, PA 18015, USA

*Corresponding Author: Wonpil Im (wonpil@lehigh.edu)

| Name | Cleaved | TMD Position | CT Truncated | Number of Atoms | Simulation Time | Number of Simulation Runs |
| --- | --- | --- | --- | --- | --- | --- |
| CH^ΔCT^ | Yes | High | Yes | 1,046,227 | 1 μs | 3 |
| CL^ΔCT^ | Yes | Low | Yes | 1,040,888 | 1 μs | 3 |
| UH^ΔCT^ | No | High | Yes | 1,080,915 | 1 μs | 3 |
| UL^ΔCT^ | No | Low | Yes | 1,080,164 | 1 μs | 3 |
| CH^CT^ | Yes | High | No | 1,080,113 | 1 μs | 3 |
| CL^CT^ | Yes | Low | No | 1,078,556 | 1 μs | 3 |
| UH^CT^ | No | High | No | 1,118,568 | 1 μs | 3 |
| UL^CT^ | No | Low | No | 1,118,375 | 1 μs | 3 |

**Table A. Simulation system information.**

|  | CH^ΔCT^1 | CH^ΔCT^2 | CH^ΔCT^3 | CL^ΔCT^1 | CL^ΔCT^2 | CL^ΔCT^3 |
| --- | --- | --- | --- | --- | --- | --- |
| A^1^ | 19% | 7%^4^ | 36% | 21% | 16% | 1% |
| B^1^ | 76% | 1% | 15% | 19% | 3% | 46% |
| C^1^ | 43% | 13% | 17% | 19% | 14% | 19% |
| A&B^2^ | 17% | 0% | 8% | 8% | 1% | 0% |
| A&C^2^ | 6% | 0% | 1% | 6% | 0% | 0% |
| B&C^2^ | 37% | 0% | 3% | 2% | 0% | 14% |
| A&B&C^3^ | 6% | 0% | 0% | 1% | 0% | 0% |

|  | UH^ΔCT^1 | UH^ΔCT^2 | UH^ΔCT^3 | UL^ΔCT^1 | UL^ΔCT^2 | UL^ΔCT^3 |
| --- | --- | --- | --- | --- | --- | --- |
| A | 5% | 32% | 2% | 3% | 9% | 5% |
| B | 35% | 38% | 41% | 2% | 28% | 2% |
| C | 12% | 16% | 53% | 45% | 3% | 33% |
| A&B | 1% | 15% | 1% | 0% | 5% | 0% |
| A&C | 1% | 9% | 1% | 1% | 1% | 2% |
| B&C | 4% | 10% | 20% | 1% | 2% | 1% |
| A&B&C | 1% | 5% | 1% | 0% | 1% | 0% |

|  | CH^CT^1 | CH^CT^2 | CH^CT^3 | CL^CT^1 | CL^CT^2 | CL^CT^3 |
| --- | --- | --- | --- | --- | --- | --- |
| A | 7% | 10% | 9% | 15% | 0% | 7% |
| B | 2% | 15% | 19% | 22% | 0% | 0% |
| C | 44% | 2% | 25% | 14% | 4% | 23% |
| A&B | 0% | 2% | 0% | 3% | 0% | 0% |
| A&C | 1% | 0% | 1% | 1% | 0% | 1% |
| B&C | 1% | 0% | 9% | 5% | 0% | 0% |
| A&B&C | 0% | 0% | 0% | 1% | 0% | 0% |

|  | UH^CT^1 | UH^CT^2 | UH^CT^3 | UL^CT^1 | UL^CT^2 | UL^CT^3 |
| --- | --- | --- | --- | --- | --- | --- |
| A | 1% | 1% | 26% | 33% | 1% | 2% |
| B | 2% | 23% | 26% | 45% | 67% | 48% |
| C | 4% | 34% | 13% | 3% | 38% | 17% |
| A&B | 0% | 1% | 5% | 14% | 1% | 0% |
| A&C | 0% | 1% | 1% | 2% | 1% | 1% |
| B&C | 0% | 9% | 3% | 2% | 29% | 1% |
| A&B&C | 0% | 1% | 0% | 1% | 1% | 0% |

**Table B. Frequency of epitope accessibility for antibody PGT128.** ^1^Three protein chains are referred to as A, B, and C. ^2^“A&B” denotes that the epitopes on both chains A and B are concurrently accessible. ^3^“A&B&C” denotes that the epitopes on all three chains are concurrently accessible. ^4^Red text highlights cases where the frequency of epitope accessibility on an individual chain is <10%. The same notations apply for Supplementary Table 3 and 4.

|  | CH^ΔCT^1 | CH^ΔCT^2 | CH^ΔCT^3 | CL^ΔCT^1 | CL^ΔCT^2 | CL^ΔCT^3 |
| --- | --- | --- | --- | --- | --- | --- |
| A | 0% | 0% | 0% | 0% | 0% | 0% |
| B | 20% | 34% | 1% | 11% | 0% | 15% |
| C | 0% | 0% | 1% | 0% | 0% | 0% |
| A&B | 0% | 0% | 0% | 0% | 0% | 0% |
| A&C | 0% | 0% | 0% | 0% | 0% | 0% |
| B&C | 0% | 0% | 0% | 0% | 0% | 0% |
| A&B&C | 0% | 0% | 0% | 0% | 0% | 0% |

|  | UH^ΔCT^1 | UH^ΔCT^2 | UH^ΔCT^3 | UL^ΔCT^1 | UL^ΔCT^2 | UL^ΔCT^3 |
| --- | --- | --- | --- | --- | --- | --- |
| A | 1% | 0% | 0% | 0% | 1% | 0% |
| B | 0% | 1% | 2% | 0% | 1% | 0% |
| C | 2% | 0% | 0% | 0% | 0% | 0% |
| A&B | 0% | 0% | 0% | 0% | 0% | 0% |
| A&C | 0% | 0% | 0% | 0% | 0% | 0% |
| B&C | 0% | 0% | 0% | 0% | 0% | 0% |
| A&B&C | 0% | 0% | 0% | 0% | 0% | 0% |

|  | CH^CT^1 | CH^CT^2 | CH^CT^3 | CL^CT^1 | CL^CT^2 | CL^CT^3 |
| --- | --- | --- | --- | --- | --- | --- |
| A | 0% | 0% | 0% | 0% | 0% | 0% |
| B | 1% | 0% | 2% | 3% | 0% | 31% |
| C | 0% | 0% | 0% | 0% | 5% | 0% |
| A&B | 0% | 0% | 0% | 0% | 0% | 0% |
| A&C | 0% | 0% | 0% | 0% | 0% | 0% |
| B&C | 0% | 0% | 0% | 0% | 0% | 0% |
| A&B&C | 0% | 0% | 0% | 0% | 0% | 0% |

|  | UH^CT^1 | UH^CT^2 | UH^CT^3 | UL^CT^1 | UL^CT^2 | UL^CT^3 |
| --- | --- | --- | --- | --- | --- | --- |
| A | 0% | 1% | 1% | 0% | 0% | 0% |
| B | 0% | 1% | 0% | 0% | 0% | 0% |
| C | 0% | 1% | 0% | 0% | 2% | 0% |
| A&B | 0% | 0% | 0% | 0% | 0% | 0% |
| A&C | 0% | 0% | 0% | 0% | 0% | 0% |
| B&C | 0% | 0% | 0% | 0% | 0% | 0% |
| A&B&C | 0% | 0% | 0% | 0% | 0% | 0% |

**Table C. Frequency of epitope accessibility for antibody PG9.**

|  | CH^ΔCT^1 | CH^ΔCT^2 | CH^ΔCT^3 | CL^ΔCT^1 | CL^ΔCT^2 | CL^ΔCT^3 |
| --- | --- | --- | --- | --- | --- | --- |
| A | 0% | 0% | 10% | 18% | 0% | 4% |
| B | 2% | 2% | 5% | 4% | 20% | 5% |
| C | 41% | 3% | 1% | 0% | 3% | 4% |
| A&B | 0% | 0% | 0% | 0% | 0% | 0% |
| A&C | 0% | 0% | 0% | 0% | 0% | 0% |
| B&C | 0% | 0% | 0% | 0% | 1% | 0% |
| A&B&C | 0% | 0% | 0% | 0% | 0% | 0% |

|  | UH^ΔCT^1 | UH^ΔCT^2 | UH^ΔCT^3 | UL^ΔCT^1 | UL^ΔCT^2 | UL^ΔCT^3 |
| --- | --- | --- | --- | --- | --- | --- |
| A | 15% | 0% | 1% | 7% | 15% | 4% |
| B | 4% | 0% | 1% | 0% | 7% | 13% |
| C | 24% | 5% | 32% | 33% | 6% | 45% |
| A&B | 0% | 0% | 0% | 0% | 2% | 0% |
| A&C | 7% | 0% | 1% | 1% | 1% | 1% |
| B&C | 0% | 0% | 0% | 0% | 0% | 10% |
| A&B&C | 0% | 0% | 0% | 0% | 0% | 0% |

|  | CH^CT^1 | CH^CT^2 | CH^CT^3 | CL^CT^1 | CL^CT^2 | CL^CT^3 |
| --- | --- | --- | --- | --- | --- | --- |
| A | 14% | 13% | 0% | 0% | 2% | 9% |
| B | 9% | 3% | 1% | 4% | 2% | 3% |
| C | 9% | 11% | 10% | 0% | 0% | 24% |
| A&B | 2% | 0% | 0% | 0% | 0% | 2% |
| A&C | 1% | 2% | 0% | 0% | 0% | 3% |
| B&C | 1% | 0% | 0% | 0% | 0% | 0% |
| A&B&C | 0% | 0% | 0% | 0% | 0% | 0% |

|  | UH^CT^1 | UH^CT^2 | UH^CT^3 | UL^CT^1 | UL^CT^2 | UL^CT^3 |
| --- | --- | --- | --- | --- | --- | --- |
| A | 17% | 0% | 16% | 4% | 22% | 5% |
| B | 6% | 0% | 1% | 0% | 0% | 0% |
| C | 57% | 19% | 57% | 35% | 14% | 44% |
| A&B | 2% | 0% | 0% | 0% | 0% | 0% |
| A&C | 6% | 0% | 11% | 2% | 2% | 0% |
| B&C | 2% | 0% | 1% | 0% | 0% | 0% |
| A&B&C | 0% | 0% | 0% | 0% | 0% | 0% |

**Table D. Frequency of epitope accessibility for antibody VRC01.**

|  | CH^ΔCT^1 | CH^ΔCT^2 | CH^ΔCT^3 | CL^ΔCT^1 | CL^ΔCT^2 | CL^ΔCT^3 |
| --- | --- | --- | --- | --- | --- | --- |
| A | 7%, 0%^1^ | 4%, 0% | 9%, 8% | 3%, 0% | 24%, 0% | 7%, 4% |
| B | 29%, 2%^2^ | 14%, 0% | 12%, 0% | 0%, 0% | 10%, 10% | 0%, 0% |
| C | 8%, 8% | 14%, 10% | 10%, 5% | 32%, 7% | 4%, 1% | 22%, 16% |
| A&B | 7%, 0% | 4%, 0% | 9%, 0% | 0%, 0% | 10%, 0% | 0%, 0% |
| A&C | 7%, 0% | 4%, 0% | 9%, 5% | 3%, 0% | 4%, 0% | 7%, 4% |
| B&C | 8%, 2% | 14%, 0% | 10%, 0% | 0%, 0% | 4%, 1% | 0%, 0% |
| A&B&C | 7%, 0% | 4%, 0% | 9%, 0% | 0%, 0% | 4%, 0% | 0%, 0% |

|  | UH^ΔCT^1 | UH^ΔCT^2 | UH^ΔCT^3 | UL^ΔCT^1 | UL^ΔCT^2 | UL^ΔCT^3 |
| --- | --- | --- | --- | --- | --- | --- |
| A | 16%, 12% | 49%, 1% | 9%, 7% | 14%, 5% | 23%, 19% | 43%, 10% |
| B | 2%, 2% | 5%, 5% | 10%, 9% | 7%, 7% | 3%, 1% | 5%, 0% |
| C | 22%, 13% | 25%, 25% | 23%, 23% | 26%, 3% | 6%, 5% | 5%, 5% |
| A&B | 2%, 2% | 5%, 1% | 9%, 7% | 7%, 5% | 3%, 1% | 5%, 0% |
| A&C | 16%, 12% | 25%, 1% | 9%, 7% | 14%, 3% | 6%, 5% | 5%, 5% |
| B&C | 2%, 2% | 5%, 5% | 10%, 9% | 7%, 3% | 3%, 1% | 5%, 0% |
| A&B&C | 2%, 2% | 5%, 1% | 9%, 7% | 7%, 3% | 3%, 1% | 5%, 0% |

|  | CH^CT^1 | CH^CT^2 | CH^CT^3 | CL^CT^1 | CL^CT^2 | CL^CT^3 |
| --- | --- | --- | --- | --- | --- | --- |
| A | 9%, 0% | 30%, 0% | 48%, 18% | 12%, 4% | 22%, 0% | 5%, 5% |
| B | 0%, 0% | 0%, 0% | 31%, 8% | 11%, 11% | 24%, 11% | 24%, 1% |
| C | 2%, 2% | 3%, 2% | 7%, 0% | 24%, 0% | 6%, 2% | 2%, 2% |
| A&B | 0%, 0% | 0%, 0% | 31%, 8% | 11%, 4% | 22%, 0% | 5%, 1% |
| A&C | 2%, 0% | 3%, 0% | 7%, 0% | 12%, 0% | 6%, 0% | 2%, 2% |
| B&C | 0%, 0% | 0%, 0% | 7%, 0% | 11%, 0% | 6%, 2% | 2%, 1% |
| A&B&C | 0%, 0% | 0%, 0% | 7%, 0% | 11%, 0% | 6%, 0% | 2%, 1% |

|  | UH^CT^1 | UH^CT^2 | UH^CT^3 | UL^CT^1 | UL^CT^2 | UL^CT^3 |
| --- | --- | --- | --- | --- | --- | --- |
| A | 5%, 4% | 18%, 16% | 33%, 0% | 31%, 0% | 31%, 0% | 41%, 0% |
| B | 26%, 16% | 8%, 5% | 11%, 7% | 2%, 1% | 2%, 0% | 18%, 1% |
| C | 25%, 0% | 7%, 0% | 20%, 9% | 3%, 2% | 29%, 15% | 7%, 7% |
| A&B | 5%, 4% | 8%, 5% | 11%, 0% | 2%, 0% | 2%, 0% | 18%, 0% |
| A&C | 5%, 0% | 7%, 0% | 20%, 0% | 3%, 0% | 29%, 0% | 7%, 0% |
| B&C | 25%, 0% | 7%, 0% | 11%, 7% | 2%, 1% | 2%, 0% | 7%, 1% |
| A&B&C | 5%, 0% | 7%, 0% | 11%, 0% | 2%, 0% | 2%, 0% | 7%, 0% |

**Table E. Frequency of epitope accessibility for antibody 35O22.** ^1^The first value represents the frequency of epitope accessibility considering only glycan shielding, and the second value represents the frequency considering the shielding of both glycan and membrane. Red text highlights cases where the first value is <10%. ^2^Magenta text highlights cases where the first value is ≥10% but the second value is <10%.

| Glycosylation Site | Composition and Type | Sequence |
| --- | --- | --- |
| N197,  N355^1^ | HexNAc(2)Hex(5)  High-mannose | 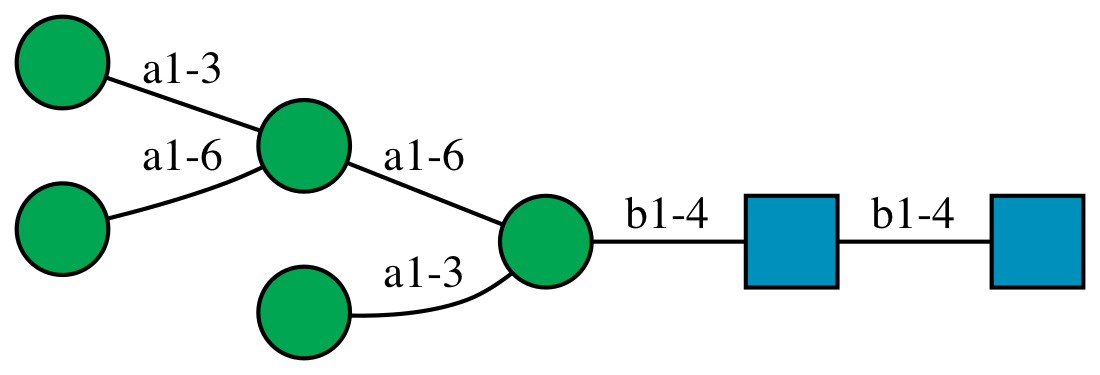 |
| N276^1^ | HexNAc(2)Hex(7)  High-mannose | 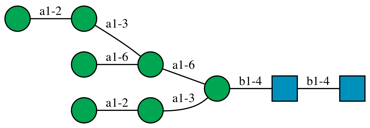 |
| N160^1^ | HexNAc(2)Hex(8)  High-mannose | 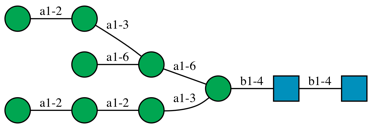 |
| N133, N156,  N234, N262,  N295, N301,  N332, N339,  N363, N386,  N392, N411,  N448 | HexNAc(2)Hex(9)  High-mannose | 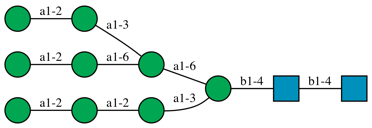 |
| N88, N137,  N185E, N185H,  N398, N406,  N618, N637^2^ | HexNAc(4)Hex(5)  Fuc(1)Neu5Ac(2)  Complex | 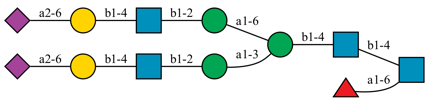 |
| N462,  N611^2,3^ | HexNAc(5)Hex(6)  Fuc(1)Neu5Ac(3)  Complex | 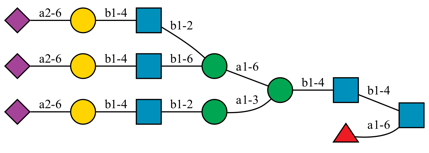 |

**Table F. Glycosylation sites, selected glycan compositions, and sequences used in this study.** ^1^The same composition can correspond to multiple possible sequences, depending on which branch the mannose (Man) is attached to. In such cases, one representative sequence was selected for structural modeling. ^2^N-Acetylneuraminic acid (Neu5Ac) can be either α2-3 or α2-6 linked to galactose (Gal). In this study, the α2-6 linkage was used for all non-reducing terminal Neu5Ac. ^3^Both the α1-3 and α1-6 branches can contain one or two antennae. Here, we added one antenna to the α1-3 branch and two antennae to the α1-6 branch.

CT-truncated (ΔCT)

| Lipid Name | Lipid Head/Tail | Number of Lipids in Leaflets | |
| --- | --- | --- | --- |
|  |  | Exoplasmic | Cytoplasmic |
| POPC | PC (16:0/18:1(9Z)) | 120 | 56 |
| PLPC | PC (16:0/18:2(9Z,12Z)) | 165 | 88 |
| PAPE | PE (16:0/20:4(5Z,8Z,11Z,14Z)) | 23 | 96 |
| POPE | PE (16:0/18:1(9Z)) | 23 | 112 |
| POPI | PI (16:0/18:1(9Z)) | 0 | 40 |
| PAPS | PS (16:0/20:4(5Z,8Z,11Z,14Z)) | 0 | 88 |
| POPA | PA (16:0/18:1(9Z)) | 0 | 8 |
| SSM | SM (d18:1/18:0) | 83 | 40 |
| NSM | SM (d18:1/24:1) | 83 | 40 |
| CMH | GlcCer (d18:1/16:0) | 30 | 0 |
| CHOL | Cholesterol | 278 | 232 |
| TOTAL |  | 805 | 800 |

Full-length (CT)

| Lipid Name | Lipid Head/Tail | Number of Lipids in Leaflets | |
| --- | --- | --- | --- |
|  |  | Exoplasmic | Cytoplasmic |
| POPC | PC (16:0/18:1(9Z)) | 120 | 49 |
| PLPC | PC (16:0/18:2(9Z,12Z)) | 165 | 77 |
| PAPE | PE (16:0/20:4(5Z,8Z,11Z,14Z)) | 23 | 84 |
| POPE | PE (16:0/18:1(9Z)) | 23 | 98 |
| POPI | PI (16:0/18:1(9Z)) | 0 | 35 |
| PAPS | PS (16:0/20:4(5Z,8Z,11Z,14Z)) | 0 | 77 |
| POPA | PA (16:0/18:1(9Z)) | 0 | 7 |
| SSM | SM (d18:1/18:0) | 83 | 35 |
| NSM | SM (d18:1/24:1) | 83 | 35 |
| CMH | GlcCer (d18:1/16:0) | 30 | 0 |
| CHOL | Cholesterol | 278 | 203 |
| TOTAL |  | 805 | 700 |

**Table G. Membrane lipid composition.**

| Stage | | Ensemble | Time Step (fs) | Total Time (ns) | Position Restraint Force Constant (kJ/mol⋅nm^2^) | | | Dihedral Restraint Force Constant (kJ/mol⋅nm^2^) |
| --- | --- | --- | --- | --- | --- | --- | --- | --- |
|  |  |  |  |  | Protein Backbone | Protein Side Chain | Lipid Head Group | Glycan Ring |
| Equilibration | 1 | NVT | 1 | 1.25 | 4000 | 2000 | 1000 | 1000 |
|  | 2 | NVT | 1 | 1.25 | 2000 | 1000 | 400 | 400 |
|  | 3 | NPT | 1 | 1.25 | 1000 | 500 | 400 | 200 |
|  | 4 | NPT | 2 | 5 | 500 | 200 | 200 | 200 |
|  | 5 | NPT | 2 | 5 | 200 | 50 | 40 | 100 |
|  | 6 | NPT | 2 | 5 | 50 | N/A | N/A | N/A |
| Production | | NPT | 4 | 1000 | N/A | N/A | N/A | N/A |

**Table H. Simulation settings for the equilibration and production stages.**
