# Supplementary material for: Conformational variability of HIV-1 Env trimer and viral vulnerability
Source: eLife. 2026 Jun 26;15:RP110107. doi: 10.7554/eLife.110107 (PMC13309128; doi:10.7554/eLife.110107)
Supplement: Peer review history

# Editor report: eLife Assessment

**DOI:** 10.7554/eLife.110107.4.sa0

**Authors:** Qiang Cui

**Affiliations:** Boston University United States

**Keywords:** Convincing, Valuable

In this **valuable** study, the authors conducted an impressive amount of atomistic simulations with a glycosylated HIV-1 envelope glycoprotein (Env) trimer in a realistic asymmetric lipid bilayer. The aim was to probe how Env transmembrane domain, cytoplasmic tail, and membrane environment influence ectodomain orientation and antibody epitope exposure. The simulations **convincingly** show that ectodomain motion is dominated by tilting relative to the membrane and explicitly demonstrate the role of membrane asymmetry in modulating the protein conformation and orientation, and the results are contextualized well in the revised version. Additional analyses of the authors' deposited MD trajectories could serve as invaluable extensions of this work to probe, for example, for exposure of cryptic epitopes and potential allosteric coupling.

# Referee report: Reviewer #3 (Public review)

**DOI:** 10.7554/eLife.110107.4.sa1

Summary:

This study uses large-scale all-atom molecular dynamics simulations to examine the conformational plasticity of the HIV-1 envelope glycoprotein (Env) in a membrane context, with particular emphasis on how the transmembrane domain (TMD), cytoplasmic tail (CT), protomer cleavage, and membrane environment influence ectodomain orientation and antibody epitope exposure. By comparing Env constructs with and without the CT, explicitly modeling glycosylation, and embedding Env in an asymmetric lipid bilayer, the authors aim to provide an integrated view of how membrane-proximal regions and lipid interactions shape Env antigenicity, including epitopes targeted by MPER-directed antibodies.

Strengths:

The authors have made a heroic effort to address the concerns raised in the first two rounds of review, and the revised manuscript is substantively improved. The addition of dynamical cross-correlation maps, expanded citation of prior computational work, clarification of the membrane composition rationale, data deposition to Zenodo, and new contextualization has improved the flow and interpretation of the manuscript throughout. Several scientifically interesting aspects of the work merit highlighting with a brief discussion on how future studies can leverage this data to build upon its impact.

A key strength of this work remains the scope, scale, and realism of the simulation systems. The authors construct a very large, nearly complete-Env-scale model that includes a glycosylated Env trimer embedded in an asymmetric bilayer, enabling analysis of membrane-protein interactions that are difficult to capture experimentally. The inclusion of specific glycans at reported sites, and the focus on constructs with and without the CT or cleavage, are well motivated by existing biological and structural data.

The observation that R696 orientation and its interacting partners give rise to asymmetric protomer conformations and distinct TMD tilts is a notable finding. The statement that interactions between R696 and lipid headgroups or CT residues can be strong enough to introduce a kink into the TMD is well-supported by representative snapshots and consistent with prior isolated-TMD simulations. The use of two initialization depths ("high" and "low") to probe R696 leaflet preference is methodologically interesting and the authors' interpretation - that there is a slight bias toward cytoplasmic leaflet interactions, but that these contacts could be highly dynamic over the course of viral entry - is appropriately cautious. It would be valuable to explicitly frame this as a hypothesis with testable predictions that future experimental or enhanced-sampling work could address. Similarly, the equilibration-driven kinking of the TMD core, consistent with prior isolated-TMD studies, represents a useful validation that extends those earlier observations to the intact trimeric context.

The simulations reveal substantial tilting motions of the ectodomain relative to the membrane, with angles spanning roughly 0-30{degree sign} (and up to ~40{degree sign} in some analyses), while the ectodomain itself remains relatively rigid. This framing, that much of Env's conformational variability arises from rigid-body tilting rather than large internal rearrangements, is an important conceptual contribution. The authors also provide interesting observations regarding asymmetric bilayer deformations, including localized thinning and altered lipid headgroup interactions near the TMD and CT, which suggest a reciprocal coupling between Env and the surrounding membrane.

The analysis of antibody-relevant epitopes across the prefusion state, including the V1/V2 and V3 loops, the CD4 binding site, and the MPER, is another strength. The study makes effective use of existing experimental knowledge in this context, for example by focusing on specific glycans known to occlude antibody binding, to motivate and interpret the simulations.

Finally, the revised text provides clear context that situates the study's findings and discrepancies within the broader literature, strengthening the manuscript's clarity and interpretability.

Future work in the field:

As the authors appropriately acknowledge within in the text, these microsecond simulations capture only the closed ground state and with limited sampling due to the already computationally intensive nature of these simulations. Their simulation setup provides interesting foundational knowledge of this state and a framework for these additional important questions.

Additionally, the authors appropriately acknowledge that CT-TMD and CT-ectodomain correlations are difficult to interpret given limited structural confidence in these regions. Future experimental and computational work in the field can extend and build upon the author's framework, particularly as the authors have made their trajectories available for the public. Re-analysis of the authors' deposited MD trajectories-such as probing for exposure of cryptic epitopes and potential allosteric coupling-could serve as valuable extensions of this work, particularly as advancements in computational analysis has reached an inflection point.

Comments on revised version.

Bravo! The improved clarity was a delight to read and will increase the impact this study has on the field.

# Author comment: Author response

**DOI:** 10.7554/eLife.110107.4.sa2

**Authors:** Yiwei Cao, Wonpil Im

**Affiliations:** Lehigh University Bethlehem United States; Lehigh University Bethlehem United States

The following is the authors’ response to the previous reviews

**Public Reviews:**

**Reviewer #1 (Public review):**

Summary:

In the manuscript "Conformational Variability of HIV-1 Env Trimer and Viral Vulnerability", the authors study the fully glycosylated HIV-1 Env protein using an all-atom forcefield. It combines long all-atom simulations of Env in a realistic asymmetric bilayer with careful data analysis. This work clarifies how the CT domain modulates the overall conformation of the Env ectodomain and characterizes different MPER-TMD conformations. The authors also carefully analyze the accessibility of different antibodies to the Env protein.

Strengths:

This paper is state-of-the-art given the scale of the system and the sophistication of the methods. The biological question is important, the methodology is rigorous, and the results will interest a broad elife audience. The authors also establish strong connections to previous literature and acknowledge the limitations of the CT-truncated protein construct, which enhances the manuscript's relevance to the community.

**Reviewer #2 (Public review):**

In this work, the authors elucidate how a viral surface protein behaves in a membrane environment and how its large-scale motions influence the exposure of antibody-binding sites. Using long-timescale, all-atom molecular dynamics simulations of a fully glycosylated, full-length protein embedded in a virus-like membrane, the study systematically examines the coupling between ectodomain motion, transmembrane orientation, membrane interactions, and epitope accessibility. Multiple model variants differing in cleavage state, initial transmembrane configuration, and presence of the cytoplasmic tail are compared to identify general features of protein-membrane dynamics relevant to antibody recognition.

A major strength of this study is the scope and ambition of the simulations. The authors perform multiple microsecond-scale simulations of a highly complex, biologically realistic system that includes the full ectodomain, transmembrane region, cytoplasmic tail, glycans, and a heterogeneous membrane. The finding that the ectodomain explores a wide range of tilt angles while the transmembrane region remains more constrained, with limited correlation between the two, offers useful conceptual insight into how global motions may be accommodated without large rearrangements at the membrane anchor. The explicit consideration of membrane and glycan steric effects on antibody accessibility further strengthens the study.

The main limitations relate to sampling and model dependence inherent to simulations of this size and complexity. The analysis of antibody accessibility is based on geometric and steric criteria, which do not capture potential conformational adaptations of antibodies or membrane remodeling during binding; the authors have appropriately noted this as a limitation.

In the revised manuscript, the authors have addressed all previously raised concerns. Time series plots of the tilt angles have been added, figure captions and visual encodings have been clarified, quantitative descriptions of angular distributions have been strengthened, and the distance metric for MPER exposure is now accompanied by temporal data. The overall presentation is substantially improved, and the conclusions are well supported by the data as presented.

**Reviewer #3 (Public review):**

Summary:

This study uses large-scale all-atom molecular dynamics simulations to examine the conformational plasticity of the HIV-1 envelope glycoprotein glycoprotein (Env) in a membrane context, with particular emphasis on how the transmembrane domain (TMD), cytoplasmic tail (CT), protomer cleavage, and membrane environment influence ectodomain orientation and antibody epitope exposure. By comparing Env constructs with and without the CT, explicitly modeling glycosylation, and embedding Env in an asymmetric lipid bilayer, the authors aim to provide an integrated view of how membrane-proximal regions and lipid interactions shape Env antigenicity, including epitopes targeted by MPER-directed antibodies.

Strengths:

The authors have made a genuine effort to address the concerns raised in the first round of review, and the revised manuscript is substantively improved. The addition of dynamical cross-correlation maps, expanded citation of prior computational work, clarification of the membrane composition rationale, data deposition to Zenodo, and the new discussion contextualizing the independence of ectodomain and TMD motions are all welcome. Several scientifically interesting aspects of the work merit highlighting before the remaining concerns are addressed.

A key strength of this work remains the scope, scale, and realism of the simulation systems. The authors construct a very large, nearly complete-Env-scale model that includes a glycosylated Env trimer embedded in an asymmetric bilayer, enabling analysis of membrane-protein interactions that are difficult to capture experimentally. The inclusion of specific glycans at reported sites, and the focus on constructs with and without the CT or cleavage, are well motivated by existing biological and structural data.

The observation that R696 orientation and its interacting partners give rise to asymmetric protomer conformations and distinct TMD tilts is a notable finding. The statement that interactions between R696 and lipid headgroups or CT residues can be strong enough to introduce a kink into the TMD is well-supported by representative snapshots and consistent with prior isolated-TMD simulations. The use of two initialization depths ("high" and "low") to probe R696 leaflet preference is methodologically interesting and the authors' interpretation - that there is a slight bias toward cytoplasmic leaflet interactions, but that these contacts could be highly dynamic over the course of viral entry - is appropriately cautious. It would be valuable to explicitly frame this as a hypothesis with testable predictions that future experimental or enhanced-sampling work could address. Similarly, the equilibration-driven kinking of the TMD core, consistent with prior isolated-TMD studies, represents a useful validation that extends those earlier observations to the intact trimeric context.

The simulations reveal substantial tilting motions of the ectodomain relative to the membrane, with angles spanning roughly 0-30° (and up to ~40° in some analyses), while the ectodomain itself remains relatively rigid. This framing, that much of Env's conformational variability arises from rigid-body tilting rather than large internal rearrangements, is an important conceptual contribution. The authors also provide interesting observations regarding asymmetric bilayer deformations, including localized thinning and altered lipid headgroup interactions near the TMD and CT, which suggest a reciprocal coupling between Env and the surrounding membrane.

The analysis of antibody-relevant epitopes across the prefusion state, including the V1/V2 and V3 loops, the CD4 binding site, and the MPER, is another strength. The study makes effective use of existing experimental knowledge in this context, for example by focusing on specific glycans known to occlude antibody binding, to motivate and interpret the simulations.

Finally, the revised discussion provides more context that situates the study's findings and discrepancies within the broader literature, strengthening the manuscript's clarity and interpretability.

Weaknesses:

The revised work is much improved, but still includes substantive issues with writing including organization, such as paragraph run-ons, and citation issues. Improving these would help readers make the most of this important study.

The revised Introduction now includes a paragraph summarizing prior MD work, which is an Improvement. However, the paragraph remains structured around the limitations and setup of previous studies (e.g., "early studies were constrained by limited computational resources", short trajectory lengths, isolated constructs) rather than their findings. Readers benefit most from understanding what those studies showed - and where the present work confirms, extends, or diverges from those results. The current framing inadvertently positions prior work as deficient scaffolding rather than as independent data points converging on shared conclusions. The Introduction could be revised to briefly summarize the key biological conclusions from prior MD studies alongside their technical context, which could then be revisited in their appropriate place alongside key results.

The authors have verified that PDB entries are cited at first mention, and this is noted. However, a recurring issue remains: key literature-supported conclusions appear in the Results and Discussion sections without accompanying citations at each point of use. Passages that summarize experimental or computational findings - particularly those used to validate or contextualize the authors' own results - require citation at every point of claim, not only at first introduction of a reference. This is not a minor stylistic preference. Downstream readers, systematic reviewers, and automated tools that map literature to claims (e.g., scite) rely on co-occurrence of claims and citations within the same passage. A citation appearing several paragraphs earlier does not carry attribution forward. As a practical example: the statement that "MPER-targeting antibodies bind effectively only after the gp120-gp41 trimer undergoes major conformational rearrangements toward a fusion-intermediate or post-fusion state (Frey et al., 2008; Alam et al., 2009; Chen et al., 2014; Lee et al., 2016)", which is appropriate. That same standard of inline attribution should be applied throughout - including in Results and Discussion subsections where prior experimental findings are mentioned without citation.

Additionally, cited literature should be framed to highlight convergence with the authors' conclusions, not primarily to limitations of previous studies. Where prior studies independently support a finding, this should be stated explicitly. Independent replication across methods and systems is one of the strongest arguments for ground truth; treating it as such would improve the manuscript's scientific standing.

Finally, the dynamical cross-correlation maps assess ectodomain-TMD coupling, and the authors appropriately acknowledge that microsecond simulations capture only the closed ground state. However, the revised manuscript does not address the question raised in the first review regarding CT-TMD and CT-ectodomain correlations. The Results section states that "very weak correlations between the ectodomain and the TMD" were found, but it is not clear whether the CT was included in this analysis or whether analogous correlation maps for CT-TMD and CT-ectodomain pairs were computed for the full-length systems. Additional analyses of the authors' deposited MD trajectories-such as probing for exposure of cryptic epitopes and potential allosteric coupling-could serve as valuable extensions of this work.

We thank the Reviewer for the further comments and suggestions. We have revised the manuscript accordingly.

The observation that R696 orientation and its interacting partners give rise to asymmetric protomer conformations and distinct TMD tilts is a notable finding. The statement that interactions between R696 and lipid headgroups or CT residues can be strong enough to introduce a kink into the TMD is well-supported by representative snapshots and consistent with prior isolated-TMD simulations. The use of two initialization depths ("high" and "low") to probe R696 leaflet preference is methodologically interesting and the authors' interpretation - that there is a slight bias toward cytoplasmic leaflet interactions, but that these contacts could be highly dynamic over the course of viral entry - is appropriately cautious. It would be valuable to explicitly frame this as a hypothesis with testable predictions that future experimental or enhanced-sampling work could address. Similarly, the equilibration-driven kinking of the TMD core, consistent with prior isolated-TMD studies, represents a useful validation that extends those earlier observations to the intact trimeric context.

At the end of the subsection “The energetically unfavorable R696 in the hydrophobic core results in asymmetric, kinked TMD conformations and disrupts membrane integrity” we have added

“Taken together, these observations suggest that interactions of R696 with lipid headgroups and CT residues may modulate TMD tilt and kink formation during viral entry. However, whether the orientation of R696 dynamically switches between the two leaflets over longer timescales and whether a preference exists for either leaflet remain to be examined in future experimental and/or enhanced sampling simulation studies.”

The revised Introduction now includes a paragraph summarizing prior MD work, which is an improvement. However, the paragraph remains structured around the limitations and setup of previous studies (e.g., "early studies were constrained by limited computational resources", short trajectory lengths, isolated constructs) rather than their findings. Readers benefit most from understanding what those studies showed - and where the present work confirms, extends, or diverges from those results. The current framing inadvertently positions prior work as deficient scaffolding rather than as independent data points converging on shared conclusions. The Introduction could be revised to briefly summarize the key biological conclusions from prior MD studies alongside their technical context, which could then be revisited in their appropriate place alongside key results.

We have modified the original fifth paragraph in the Introduction section and subdivided it into two separate paragraphs to emphasize the key biological conclusions in prior simulation studies.

“Molecular dynamics (MD) simulations have been employed to investigate the stability and conformational properties of monomeric and trimeric TMD. An early study of the trimeric TMD established a foundational understanding of the domain's stability, though it was limited by the computational resources available at the time (Kim et al., 2009). Subsequent work utilizing metadynamics found that the monomeric TMD is characterized by significant conformational plasticity and multiple metastable states, with the individual helix tilting in the bilayer and the midspan arginine (R696) interacting with lipid headgroups in either leaflet (Gangupomu et al., 2010; Baker et al., 2014). Baker et al. also simulated the monomeric TMD on Anton supercomputers, extended sampling to the multi-microsecond time scale, and demonstrated that TMD tilting and the interaction of R696 with lipids lead to local membrane thinning and water defects (Baker et al., 2014). Hollingsworth et al. modeled and simulated trimeric TMD in an asymmetric membrane and observed that TMD tilting and membrane thinning also occurred for the trimeric helical bundle, where water and ions permeated to stabilize the three positively charged R696 residues (Hollingsworth et al., 2018).

Piai et al. determined the NMR structure of a construct comprising the MPER, TMD, and CT, which currently serves as the only PDB structure to include the majority of the CT residues. They complemented this structural work with MD simulations to assess the structural stability of the trimeric MPER–TMD–CT complex (Piai et al., 2021). Recently, Majumder et al. simulated the same MPER–TMD–CT complex and applied a machine learning-based approach to classify the diverse conformational ensemble of the MPER-TMD-CT (Majumder et al., 2025). Maillie et al. combined conventional MD, steered MD, and coarse-grained simulations to demonstrate that interactions between MPER-targeting antibodies and membrane lipids are critical for effective epitope recognition (Maillie et al., 2025). In addition, MD simulations have been extensively applied to characterize the well-studied ectodomain.”

The authors have verified that PDB entries are cited at first mention, and this is noted. However, a recurring issue remains: key literature-supported conclusions appear in the Results and Discussion sections without accompanying citations at each point of use. Passages that summarize experimental or computational findings - particularly those used to validate or contextualize the authors' own results - require citation at every point of claim, not only at first introduction of a reference. This is not a minor stylistic preference. Downstream readers, systematic reviewers, and automated tools that map literature to claims (e.g., scite) rely on co-occurrence of claims and citations within the same passage. A citation appearing several paragraphs earlier does not carry attribution forward. As a practical example: the statement that "MPER-targeting antibodies bind effectively only after the gp120-gp41 trimer undergoes major conformational rearrangements toward a fusion-intermediate or post-fusion state (Frey et al., 2008; Alam et al., 2009; Chen et al., 2014; Lee et al., 2016)", which is appropriate. That same standard of inline attribution should be applied throughout - including in Results and Discussion subsections where prior experimental findings are mentioned without citation.

Additionally, cited literature should be framed to highlight convergence with the authors' conclusions, not primarily to limitations of previous studies. Where prior studies independently support a finding, this should be stated explicitly. Independent replication across methods and systems is one of the strongest arguments for ground truth; treating it as such would improve the manuscript's scientific standing.

In addition to summarizing the biological conclusions from prior simulation studies in our response to the previous comment, we have also added the following citations.

“Human immunodeficiency virus type 1 (HIV-1) is the most prevalent strain of HIV responsible for the development of acquired immunodeficiency syndrome (AIDS) (Sharp et al., 2011). The HIV-1 envelop (Env) consists of a host cell-derived lipid membrane and viral glycoproteins that play a crucial role in mediating viral entry into host cells. The Env glycoprotein is initially synthesized in the endoplasmic reticulum (ER) as a precursor gp160 and cleaved by furin into two subunits, gp120 and gp41. The non-covalently associated gp120–gp41 complex is transported to the cell surface in the form of a trimer, where it is subsequently incorporated into the envelope of nascent virions during viral assembly (Wyatt et al., 1998). The exposure of Env protein is essential for binding to the primary receptor CD4 and the co-receptors CCR5 or CXCR4, triggering membrane fusion and viral entry (Dalgleish et al., 1984; Feng et al., 1996; Huang et al., 1996). However, this exposure also renders the virus susceptible to immune attack. In response to host immune pressure, Env is densely coated with N-linked glycans added during post-translational modification in the ER and Golgi apparatus, which effectively shield vulnerable epitopes from immune recognition (Wei et al., 2003).”

“While MPER plasticity has been linked to its role in virus-host membrane fusion because it enables the ectodomain and TMD to adopt distinct orientations during large-scale structural rearrangements (Salzwedel et al., 1999), our results show that this flexibility is already inherently present in the prefusion state.”

“However, transition among these three states occur on millisecond-to-second timescales (Munro et al., 2014).”

Finally, the dynamical cross-correlation maps assess ectodomain-TMD coupling, and the authors appropriately acknowledge that microsecond simulations capture only the closed ground state. However, the revised manuscript does not address the question raised in the first review regarding CT-TMD and CT-ectodomain correlations. The Results section states that "very weak correlations between the ectodomain and the TMD" were found, but it is not clear whether the CT was included in this analysis or whether analogous correlation maps for CT-TMD and CT-ectodomain pairs were computed for the full-length systems. Additional analyses of the authors' deposited MD trajectories-such as probing for exposure of cryptic epitopes and potential allosteric coupling-could serve as valuable extensions of this work.

We have updated the manuscript to address the correlations involving the CT. Figure 2—figure supplements 12 and 13 display the dynamical cross-correlation maps (DCCM) for the full-length systems (including the CT), which indicate low correlations between the ectodomain and the CT. We have modified the figure captions to explicitly state that the CT is included in these analyses. We have also clarified in the text that we do not further interpret the coupling of the CT with the other domains. As the Reviewer noted, the high structural heterogeneity of the CT makes defining consistent parameters (such as a tilt angle) impractical. Given this variability, along with the inherent uncertainty in the experimental structure of the CT, we believe it is important to avoid over interpreting these observations.

“Although Figure 2—figure supplements 12 and 13 also show low correlations between the ectodomain and the CT, we do not further interpret the coupling of the CT with the other domains due to its structural heterogeneity and the inherent uncertainty in its experimental structure.”

We have modified captions of Figure 2—figure supplements 10–13

**Recommendations for the authors:**

**Reviewer #3 (Recommendations for the authors):**

The authors have made meaningful progress in addressing first-round concerns. The remaining issues center on how prior literature is framed and integrated - not just cited - throughout the manuscript, consistent attribution at each point of claim, clarification of the CT correlation analysis, and major writing improvements. Addressing these points would substantially strengthen the manuscript's contribution to the field.

Abstract

"knowledge of the cytoplasmic tail (CT) is virtually absent" is overstated. While structural data for the CT are limited and largely uncertain, the CT has been extensively studied functionally and some NMR structural data exist (Piai et al., 2021; Murphy et al., 2017). Suggest revising to reflect that high-resolution structural information for the CT in the context of the intact trimer remains limited

We have revised the abstract according to the Reviewer’s suggestion.

“While structural information is available for the membrane-proximal external region (MPER) and transmembrane domain (TMD), these regions remain comparatively understudied. Furthermore, high-resolution structural information for the cytoplasmic tail (CT), particularly within the context of the intact trimer, is limited and largely uncertain.”

Introduction

The first paragraph is unreferenced. Foundational claims about HIV-1 biology, Env processing, and glycan shielding should carry at least landmark citations for readers new to the field.

We have added references to the first paragraph.

“Human immunodeficiency virus type 1 (HIV-1) is the most prevalent strain of HIV responsible for the development of acquired immunodeficiency syndrome (AIDS) (Sharp et al., 2011). The HIV-1 envelop (Env) consists of a host cell-derived lipid membrane and viral glycoproteins that play a crucial role in mediating viral entry into host cells. The Env glycoprotein is initially synthesized in the endoplasmic reticulum (ER) as a precursor gp160 and cleaved by furin into two subunits, gp120 and gp41. The non-covalently associated gp120–gp41 complex is transported to the cell surface in the form of a trimer, where it is subsequently incorporated into the envelope of nascent virions during viral assembly (Wyatt et al., 1998). The exposure of Env protein is essential for binding to the primary receptor CD4 and the co-receptors CCR5 or CXCR4, triggering membrane fusion and viral entry (Dalgleish et al., 1984; Feng et al., 1996; Huang et al., 1996). However, this exposure also renders the virus susceptible to immune attack. In response to host immune pressure, Env is densely coated with N-linked glycans added during post-translational modification in the ER and Golgi apparatus, which effectively shield vulnerable epitopes from immune recognition (Wei et al., 2003).”

A paragraph break after "... and cytoplasmic tail (CT), are relatively understudied" would improve readability by separating the general context from the MPER/TMD-specific discussion that follows.

A paragraph break before "Similarly, there are different conclusions about" would separate the TMD oligomeric state discussion from the MPER conformation discussion and improve navigation.

A paragraph break after "Despite these advances, it remains challenging to investigate the gp120-gp41 trimer as an intact entity considering its structural complexity" would clearly delineate the literature context from the description of the present work.

We have introduced paragraph breaks as suggested to improve the flow and readability of the introduction.

The biological rationale for simulating both cleaved and uncleaved systems should be stated explicitly in the Introduction. Readers unfamiliar with the furin cleavage biology and NFL trimer constructs will benefit from a sentence explaining why this comparison is informative.

In the middle of the last paragraph in the Introduction section we have added

“While host furin cleavage of the gp160 precursor into gp120 and gp41 is a prerequisite for viral infectivity (McCune et al., 1988), native virions also incorporate a fraction of uncleaved gp160 (Zhang et al., 2021). Furthermore, many current immunogen designs, such as NFL and UFO constructs, utilize a covalent linker to stabilize the metastable prefusion conformation (Sharma et al., 2015; Kong et al., 2016). Therefore, we simulated both cleaved and uncleaved trimers to explore how the absence of proteolytic cleavage impacts the conformational landscape.”

The modifier “subsequently” in “Majumder et al. subsequently simulated...” implies temporal sequence and invites the inference that Majumder et al.'s work is less sophisticated or prior. Given that both works are recent and peer-reviewed, a neutral modifier such as “recently” or “independently” is more appropriate.

We agree that a more neutral modifier is appropriate and have replaced “subsequently” with “recently” to avoid any unintended inference.

In the beginning of the sixth paragraph in the Introduction section we have modified

“Piai et al. determined the NMR structure of a construct comprising the MPER, TMD, and CT; to date, this is the only PDB structure including the majority of CT residues. They complemented this structural work with MD simulations to access the structural stability of the trimeric MPER–TMD–CT complex (Piai et al., 2021). Recently, Majumder et al. simulated the same MPER–TMD–CT complex and applied a machine learning-based approach to classify its conformational ensemble (Majumder et al., 2025).”

The sentence "Moreover, we selected several bNAbs targeting the epitopes across different regions of the Env protein and demonstrate that the simulation trajectories can be used to assess the epitope accessibility" implies that simulations of antibody binding were performed. This should be rephrased, for example: "Moreover, we selected various epitopes across Env that are targeted by bNAbs and demonstrate that the MD simulation trajectories can be used to assess epitope accessibility."

At the end of the Introduction section we have modified

“Moreover, by analyzing epitopes targeted by various bNAbs, we demonstrate that the simulation trajectories can be leveraged to assess the epitope accessibility.”

The revised Methods section now cites van Meer et al. (2008) and Sampaio et al. (2011) as primary experimental sources for plasma membrane composition, which is appropriate. However, the Introduction still contains the statement: "we built a model of full-length gp120-gp41 trimer embedded in a lipid bilayer mimicking the lipid composition of the mammalian plasma membrane (Pogozheva et al., 2022)". This cites only the authors' own prior simulation study. A primary experimental reference (van Meer et al., 2008 and/or Sampaio et al., 2011) should be added here as well, so that readers encountering the claim in the Introduction have direct access to the supporting evidence.

At the beginning of the last paragraph in the Introduction section we have modified

“In this work, we built a model of full-length gp120–gp41 trimer embedded in a lipid bilayer mimicking the lipid composition of the mammalian plasma membrane (van Meer et al., 2008; Sampaio et al., 2011; Ingolfsson et al., 2014; Pogozheva et al., 2022) (Figure 1).”

Additionally, a brief note in the Introduction on the cell type specificity of the plasma membrane model used (or its absence) would be informative, as membrane composition varies substantially across mammalian cell types and the choice has potential consequences for the conclusions.

We have added a brief note clarifying that differences between the model membrane and the native viral envelope may influence the study's conclusions, particularly regarding protein-lipid interactions.

“We chose this composition as a representative baseline, though we acknowledge that the native viral envelope may exhibit a distinct lipid profile that could influence protein-lipid interactions.”

Results

Connecting the observed accessibility frequencies to known neutralization potency, breadth, or escape propensity for each antibody class (PGT128, PG9, VRC01, 35O22, 10E8, 4E10) would provide a mechanistic framework and substantially increase the impact of this section. Even a brief discussion of how glycan shielding dynamics relate to reported neutralization sensitivity data would add value.

We have expanded the Results section to include a comparison between our computational accessibility frequencies and established experimental metrics (potency and breadth).

At the end of each paragraph in the subsection “Ectodomain epitopes are conditionally accessible, whereas MPER epitopes are virtually inaccessible in the closed prefusion state” we have added

“The high accessibility frequency observed for the PGT128 epitope aligns with its exceptional potency. As demonstrated by Walker et al., PGT128 is capable of neutralizing approximately 72% of global isolates with a median *IC*_50_ of ~0.02 µg/mL. This potency is approximately 10-fold greater than that of PG9 and VRC01, though its breadth is lower than the 93% reported for VRC01 (Walker et al., 2011). This comparatively lower breadth may be attributed to strict sequence dependency. Because PGT128 recognition depends on the N332-centered glycan epitope, loss, truncation, or shifting of the N332 glycan to N334 prevents productive engagement regardless of local steric accessibility.”

“This is consistent with the lower neutralization potency and moderate breadth of PG9, which exhibits a median *IC*_50_ of ~0.22 µg/mL and a breadth of ~79% (Walker et al., 2009).”

“This intermediate accessibility is consistent with the biological requirement of the CD4 binding site to remain periodically available for receptor engagement while maintaining a certain degree of glycan shielding to evade neutralization. The potency of VRC01 is even lower than that of PG9, with a reported median *IC*50 of ~0.32 µg/mL, but it possesses an exceptionally high breadth of ~93% (Wu et al., 2010; Walker et al., 2011).”

“Altogether, these results demonstrate that epitope accessibility for this antibody is highly sensitivity to the membrane environment, glycan orientation and ectodomain tilting. This complex dependency provides a structural context for the experimental profile of 35O22, which exhibits high potency with a median *IC*_50_ of ~0.03 µg/mL, but a relatively limited breadth of ~62% (Huang et al., 2014).”

“Though differing in potency — with 10E8 exhibiting a median *IC*_50_ of ~0.35 µg/mL compared to ~1.93 µg/mL for 4E10 — both antibodies demonstrate extremely high breadth of ~98% (Huang et al., 2012). This extensive breadth is primarily attributed to the high sequence conservation of the MPER across global isolates. The negligible epitope accessibility observed in the prefusion trimer supports the conclusion that these antibodies require the transition of the Env trimer into intermediate states to fully engage their epitopes (Frey et al., 2008).”

The first paragraph of the Results section dives directly into trajectory notation without a brief summary of the simulation systems. A short opening paragraph (2-3 sentences) summarizing the number of systems, the variables tested (cleavage, CT presence, TMD position), and the total number of trajectories would orient the reader before the naming convention is introduced.

We have moved the original first sentence in the Material and methods — Simulation details subsection to the beginning of the Results section. This sentence summarizes all the configurations we have considered and the number of independent trajectories for each configuration.

“The combination of cleavage state (cleaved vs. uncleaved), sequence length (full-length vs. CT-truncated), and initial TMD position in the membrane (high vs. low) resulted in eight distinct configurations, and we performed three independent 1-μs all-atom MD simulations for each configuration.”

The statement "very weak correlations between the ectodomain and the TMD" leaves open the question of CT-TMD and CT-ectodomain correlations. If a tilt angle cannot be defined for the CT due to its structural heterogeneity, this should be stated.

We have updated the manuscript to address the correlations involving the CT. Figure 2—figure supplements 12 and 13 display the dynamical cross-correlation maps (DCCM) for the full-length systems (including the CT), which indicate low correlations between the ectodomain and the CT. We have modified the figure captions to explicitly state that the CT is included in these analyses. We have also clarified in the text that we do not further interpret the coupling of the CT with the other domains. As the Reviewer noted, the high structural heterogeneity of the CT makes defining consistent parameters (such as a tilt angle) impractical. Given this variability, along with the inherent uncertainty in the experimental structure of the CT, we believe it is important to avoid over interpreting these observations.

“Although Figure 2—figure supplements 12 and 13 also show low correlations between the ectodomain and the CT, we do not further interpret the coupling of the CT with the other domains, considering its structural heterogeneity and the inherent uncertainty in its experimental structure.”

We have modified captions of Figure 2—figure supplements 10–13

Throughout the Results, several long paragraphs could be broken up. In particular, the TMD section and the MPER exposure section each contain dense multi-example run-on paragraphs that would benefit from subdivision.

We agree with the Reviewer and have introduced multiple paragraph breaks in the Results section to improve the flow and readability. In instances where longer paragraphs remain, they have been intentionally preserved to maintain the logical integrity of closely linked results, ensuring the reader can follow a single cohesive argument without interruption.

Discussion

The statement "transition among three states occur on millisecond-to-second timescales" is an important claim that contextualizes the limitations of the microsecond simulations, but it is currently uncited. This should be attributed to the relevant experimental smFRET work (Munro et al., 2014 is cited in the preceding sentence, but not explicitly for this claim) and/or any additional literature that established these timescales for Env conformational switching.

We have now explicitly attributed the claim regarding the millisecond-to-second timescales of Env conformational transitions to the relevant smFRET literature (Munro et al., 2014).

In the middle of the second paragraph in the Discussion section we have added

“However, transition among these three states occur on millisecond-to-second timescales (Munro et al., 2014).”

The Discussion contains several extended paragraphs that could be subdivided to improve readability and help the reader navigate between distinct topics (e.g., MPER flexibility, CT effects, coupling, lipid composition, antibody accessibility).

We have subdivided the Discussion section as suggested by the Reviewer to improved readability.
